# Sulfasalazine, a potent suppressor of gastric cancer proliferation and metastasis by inhibition of xCT: Conventional drug in new use

**DOI:** 10.1111/jcmm.16548

**Published:** 2021-05-14

**Authors:** Jinfu Zhuang, Xing Liu, Yuanfeng Yang, Yiyi Zhang, Guoxian Guan

**Affiliations:** ^1^ Department of Colorectal Surgery The First Affiliated Hospital of Fujian Medical University Fuzhou China

**Keywords:** gastric cancer, growth, metastasis, sulfasalazine, xCT

## Abstract

The aim of this study was to explore the role of sulfasalazine on proliferation and metastasis in gastric cancer by inhibition of xCT. The relationships between clinical characteristics and xCT expression were analysed. An immunohistochemical staining assay and Western blot were performed among gastric cancers and normal gastric tissues. qPCR and Western blot were also used to evaluate the mRNA and protein expression in the normal gastric cell and eight gastric cancer cells, respectively. CCK‐8 and colony formation assays were used to evaluate the effect of sulfasalazine on the proliferation and colony formation ability of three gastric cancers. The effect of sulfasalazine on the migration and invasion abilities of three cancer cells was assessed by the Transwell assay. xCT protein is up‐regulated in gastric cancer specimens and cells. Three gastric cancer cells with high, medium and low expression of xCT were selected for the following analyses. CCK‐8 assays revealed that sulfasalazine could attenuate the proliferation of HGC‐27 and AGS. Also, the colony formation assay revealed that sulfasalazine might attenuate the colony formation ability in HGC‐27 and AGS cells. Plus, the Transwell assays demonstrated that sulfasalazine might attenuate the migration and invasion abilities in HGC‐27 and AGS cells. In conclusion, higher expression of xCT is associated with advanced tumour stage and poor overall survival of gastric cancer. Sulfasalazine can attenuate the proliferation, colony formation, metastasis and invasion of gastric cancer in vitro. Further study is required to validate our findings.

## 1 INTRODUCTION

Gastric cancer remains a global health problem, with more than one million newly diagnosed gastric cancer patients each year worldwide.^1^, ^2^ It has been reported by Bray et al that there were 1 033 701 new cases and 782 685 deaths related to gastric cancer in 2018. The advanced stage, when diagnosed and the unsatisfactory prognosis because of the high recurrence rate, makes gastric cancer accompany poor five‐year survival.^4^ The standard therapy for curative gastric cancers remains the surgery involved complete resection with standardized D2 lymphadenectomy.^5^ However, advanced gastric cancers with distant metastasis are usually incurable.^6^ Therefore, alternative therapeutic options are usually and urgently needed.

System x_c_^−^ has been reported to play an essential role in capturing cysteine from the extracellular environment. The cysteine is the basic raw material for intercellular glutathione synthesis. xCT plays a predominant role in the System x_c_^−^; therefore, the intracellular glutathione levels are determined by the expression and function of xCT. Accumulated evidence has demonstrated that xCT plays a crucial role in many cancers, like bladder cancer,^7^ thyroid cancer,^8^ triple‐negative breast cancer^9^ and prostate cancer.^10^ However, the role of the xCT in gastric cancer has not been well elucidated yet.

Sulfasalazine, a conventional drug, which is widely utilized in treating inflammatory diseases and rheumatoid arthritis, can also inhibit the expression and function of xCT.^10^ Kim et al^11^ demonstrated that sulfasalazine could induce ferroptotic cell death in head and neck cancer. Garcia et al^12^ revealed that sulfasalazine could promote human glioblastoma cell death through the intracellular oxidative response imbalance. Also, sulfasalazine could play a critical role in regulating various tumours like hepatocellular carcinoma^13^ and breast cancer.^14^ Whether the sulfasalazine can attenuate the oncological behaviour of gastric cancer through inhibiting xCT remains unknown. Therefore, the effect of sulfasalazine on proliferation, colony formation, metastasis and invasion of gastric cancer is required.

In this study, the mRNA expression and protein expression of xCT in human gastric cancer tissues and normal gastric tissues were evaluated. Also, the mRNA and protein expression of xCT in eight different gastric cancer cell lines was evaluated. Then, we utilized a conventional drug, sulfasalazine, to attenuate the expression of xCT to evaluate the effect of sulfasalazine on proliferation, colony formation, metastasis and invasion of gastric cancer.

## 2 METHODS

### 2.1 Clinical specimens

All gastric tissue specimens were obtained from the First Affiliated Hospital of Fujian Medical University. Informed consents were conducted, and written consents were obtained before the operation.

### 2.2 Clinicopathological characteristics

A total of 136 patients were included in this study. The inclusion criteria were as follows: (a) age between 18‐70 years old; (b) gastric cancer was pathologically confirmed. The exclusion criteria were as follows: (a) concomitant with other malignant diseases or other malignant diseases within 5 years; (b) having a history of continuous systemic corticosteroids or immunosuppressive drugs; (c) patients with incomplete clinicopathological data. The clinicopathological parameters were collected, including age, gender, tumour location, tumour size, the grade of differentiation, depth of invasion, lymphatic metastasis, pTNM stage, vascular invasion and never invasion.

### 2.3 Immunohistochemistry assay

A total of 136 patients with gastric adenocarcinoma who underwent surgery and pathologically confirmed at the First Affiliated Hospital of Fujian Medical University were included in this study. Immunohistochemistry assay was performed to validate the expression of xCT between gastric cancer and normal gastric tissues. Briefly, formalin‐fixed and paraffin‐embedded gastric cancer tumour specimens and normal gastric tissues were prepared and sectioned. After dewaxing, hydration and antigen retrieval, the immunohistochemical staining was conducted by utilizing the rabbit monoclonal antibody against human xCT (1:1000, ab37185; Abcam). Finally, the staining of the slices was confirmed by a senior pathologist under the microscope after DAB staining, haematoxylin counterstaining and neutral resin sealing.

### 2.4 Cell culture

Human gastric cancer cell lines NCI‐N87, SGC‐7901, Mkn‐74, NUGC‐3, Mkn‐45, MGC‐803, AGS and HGC‐27, were obtained from the cell bank of the Chinese Academy of Sciences (Shanghai, China). These cell lines were cultured and maintained in DMEM (Gibco BRL) with 10% foetal bovine serum (FBS) and 1% penicillin‐streptomycin and incubated at 37°C and 5% CO2. For drug treatment CCK‐8 assays, cells were treated with a final concentration of 0, 200, 400, 800 and 1000 mmol L^−1^ sulfasalazine (Absin, Shanghai, China).

### 2.5 RT‐qPCR

RT‐qPCR was utilized to evaluate the mRNA expression of xCT. Total RNA was extracted from gastric cancer cell lines by Qiagen RNeasy kit (Qiagen Bioinformatics). We then utilized an RT Reagent kit (Takara Bio Inc.) to reverse transcription of RNA into cDNA. The primers (BioSune Biotechnology Co., Ltd.) of xCT were as follows: xCT‐F: AGCACATAGCCAATGGTGAC, xCT‐R: GCTGGCTGGTTTTACCTCAA, β‐actin‐F: CCTGGCACCCAGCACAAT, β‐actin‐R: GGGCCGGACTCGTCATACT. The primers were designed to amplify cDNA with SYBR Premix EX Taq kit (Takara Bio Inc.) The PCR conditions were 95°C for 2 minutes, 95°C for 15 seconds and 60°C for 30 seconds for 40 cycles. The relative expression of xCT mRNA was normalized to that of β‐actin. The $2^{‐\Delta \Delta \text{Cq}}$ method was used to calculate the relative expression of xCT mRNA.^15^

### 2.6 Western blot analysis

We utilized cell lysis buffer (Beyotime), containing 1% PMSF (Amresco), to cleave proteins for 30 minutes on ice. Then, we centrifuged the lysed cells at 12 000 *g* for 10 minutes at 4°C to extract the supernatant for protein quantification. We utilized the BCA Protein Assay Kit (Thermo Fisher Scientific, Inc.) to quantify protein concentration. The obtained supernatant was then boiled for 10 minutes by adding 5X SDS. Protein (50 μg) was added to the prepared 12% SDS‐PAGE gels for electrophoretic separation and transferred to 0.45 µm PVDF membranes (Amersham Hybond, GE Healthcare). We then utilized 1% albumin from bovine serum (Amresco) to block the PVDF membranes for 2 hours. Then, the membranes were incubated overnight with diluted xCT (1:1000, ab37185; Abcam) and β‐actin (1:1000, ab179467; Abcam) antibodies on a shaker at 4°C. We washed the membranes with TBS‐T (0.1% Tween‐20) at room temperature three times for 10 minutes. The goat anti‐rabbit IgG H&L (HRP) (1:2000, ab7090; Abcam) for 1 hour was utilized to incubate the membranes. After washed, the membranes were exposed to enhanced chemiluminescence substrate detection solution (Lulong Biotech) subsequently.

### 2.7 Cell proliferation assay

CCK‐8 (Dojindo) was utilized to evaluate cell proliferation. Cells were seeded in a 96‐well plate. The density is 1000 cells/well. Cells were then incubated at 37°C, 5% CO_2_. 100 μL of CCK‐8 serum‐free medium was added at 24, 48, 72 and 96 hours after discarding the culture solution. We estimated cell proliferation by using a microplate reader (BioTek) after 1 hour of incubation.

### 2.8 Colony formation assay

We seeded the cells (500/well) in a 6‐cm plate. We utilized a culture medium containing 10% FBS, and cells were cultured for 14 days. The cells were infiltrated with methanol and stained with crystal violet after discarding the culture solution for 10 minutes. After that, cells were washed with water and then dried and counted.

### 2.9 Cell migration and invasion assay

Gastric cancer cells were cultured in a serum‐free medium, and then, we utilized 4 × 10^4^ gastric cancer cells in the migration assay. Firstly, gastric cancer cells were seeded into the upper chamber of a Transwell insert (8‐mm pore size; Corning Inc.). Second, we added a medium with 20% FBS as a chemoattractant in the lower chamber. Matrigel (BD Bioscience) was coated on the upper chamber in the invasion assay. We seeded 9 × 10^4^ cells in the upper chamber and added a medium containing 20% FBS in the lower chamber. We incubated the Transwell chamber at 37°C, 5% CO_2_ for 48 hours. After that, we discarded the medium and washed the well by utilizing calcium‐free PBS. Then, we utilized methanol to fix the cells for 30 minutes and stain the cells with 0.1% crystal violet for 20 minutes. We gently wiped off the un‐migrated cells in the upper chamber with a cotton swab. Finally, these un‐migrated cells were counted under the microscope.

### 2.10 Statistical analysis

We utilized GraphPad Prism 7 software to perform statistical analyses. The data were presented as the means ± standard deviation (SD) and analysed by one‐way ANOVA or Student's *t* test. *P* < .05 was considered to be statistically significant.

## 3 RESULTS

### 3.1 Clinical characteristics and the differentially expressed xCT in gastric cancer

The clinicopathological features of gastric cancer patients with differently expressed xCT are demonstrated in Table 1. There was no significant difference in age, gender, location, tumour size, the grade of differentiation, lymphatic metastasis, vascular invasion and nerve invasion between the low xCT expression and high xCT expression groups. Nevertheless, in terms of invasion depth, the high xCT expression group has more T3 and T4 patients (*P* = .024). Plus, when it comes to the pTNM stage, the high xCT expression group has more stage III and stage IV patients than the low xCT expression group (*P* = .027).

**TABLE 1:** Clinicopathological parameters of GC patients with different xCT expression

Clinicopathological parameters	No of patients	xCT	Expression	*X^2^*	*P*
		Lower	Higher		
Age (y)				1.580	.209
≤60	51	21	30		
>60	85	26	59		
Gender				0.102	.750
Male	99	35	64		
Female	37	12	25		
Location				4.201	.122
Upper	37	14	23		
Middle	33	19	24		
Lower	56	14	42		
Tumour size (cm)				0.256	.613
≤5	77	28	49		
>5	59	19	40		
Grade of differentiation				0.106	.745
Poor and not	96	34	62		
Well and moderate	40	13	27		
Depth of invasion				5.090	.024*
T1, T2	9	0	9		
T3, T4	127	47	80		
Lymphatic metastasis				2.958	.398
N0	23	6	17		
N1	29	9	20		
N2	25	7	18		
N3	59	25	34		
pTNM stage				4.888	.027*
I, II	29	5	24		
III, IV	107	42	65		
Vascular invasion				0.078	.780
Yes	63	21	42		
No	73	26	47		
Nerve invasion				2.696	.260
Yes	46	18	28		
No	89	28	61		

* *P* < .01, statistical significance.

### 3.2 The immunohistochemical staining confirms the expression of xCT

**FIGURE 1:**
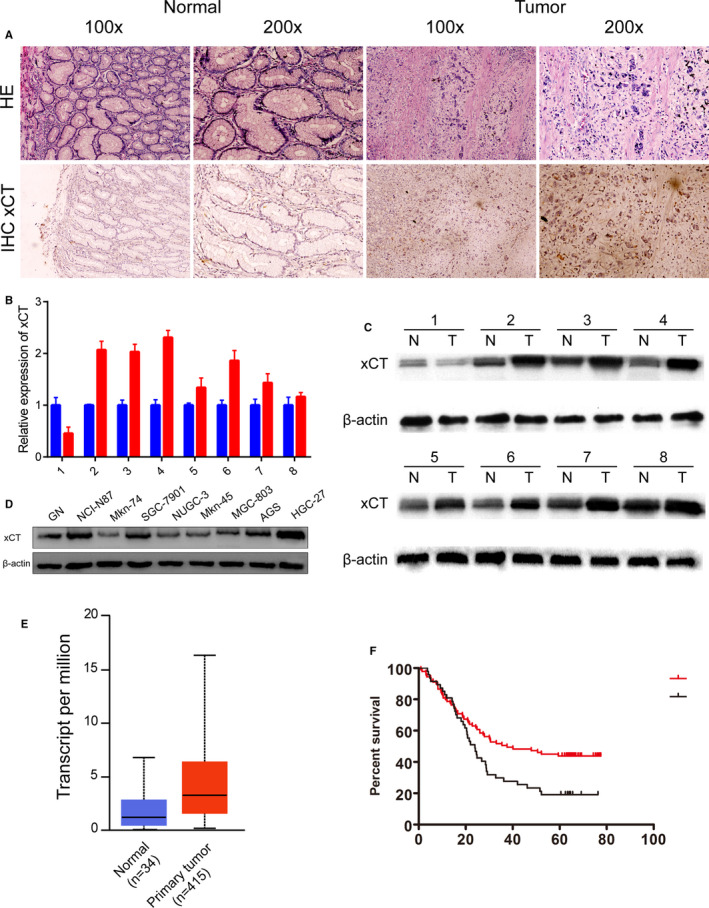
mRNA and protein expression of xCT in gastric cancer clinical samples and gastric cancer cell lines. A, The upper panel demonstrates the HE staining of normal and tumour tissues at 100× and 200× magnification. The lower panel demonstrates the immunochemical staining of the expression of xCT in gastric cancer tissues and normal gastric cancer tissues at 100× and 200× magnification. B, qPCR demonstrated the relative expression of xCT in eight paired gastric cancer and normal gastric tissues. C, The protein expression of xCT in eight gastric cancer specimens and paired corresponding normal gastric tissues. D, The protein expression of xCT in eight gastric cancer cell lines and normal gastric cells. E, The mRNA expression of xCT in tumour and normal gastric tissues based on data from the TCGA database. F, Survival analysis demonstrated that higher expression of xCT is associated with poor overall survival (*P* < .05)

The haematoxylin‐eosin (HE) staining demonstrates the morphological differences between gastric cancers and normal gastric tissues. An immunohistochemical staining assay was performed among gastric cancers and normal gastric tissues. The results demonstrated that xCT is differentially expressed between gastric cancers and normal gastric tissues. The immunohistochemical staining results demonstrated that the expression of xCT was up‐regulated in gastric cancer compared with normal gastric tissues (Figure 1A).

### 3.3 xCT protein is up‐regulated in gastric cancer specimen

A total of eight paired gastric cancer tissues and corresponding normal gastric tissues were compared. The qPCR results demonstrated that xCT is up‐regulated in gastric cancer compared with normal gastric tissues (Figure 1B). The Western blot results demonstrated that xCT is up‐regulated in the tumour groups compared with the normal groups (Figure 1C), consistent with the qPCR results. We then performed the Western blot assays in GN (normal gastric) cells and eight different gastric cancer cells, including NCI‐N87, SGC‐7901, Mkn‐74, NUGC‐3, Mkn‐45, MGC‐803, AGS and HGC‐27. The Western blot results demonstrated that the protein expression of xCT is up‐regulated in NCI‐N87, SGC‐7901, AGS, MGC‐803 and HGC‐27 cell lines compared with GN cells. There was no significant difference in the expression of xCT among and NUGC‐3 and Mkn‐45 cells. However, the expression of xCT is down‐regulated in Mkn‐74 cells (Figure 1D). The expression of xCT in gastric cancer was validated by the expression profiles in the TCGA database. The results demonstrated that the expression of xCT is up‐regulated in gastric cancer tissues compared with normal gastric tissues, which is consistent with our study (Figure 1E). The higher expression of xCT is associated with a lower overall survival rate in patients who underwent surgery (Figure 1F). The protein expression of xCT in HGC‐27 is relatively higher among gastric cancer cells, and the expression of xCT in MGC‐803 is relatively lower among gastric cancer cells. Plus, the protein expression of xCT in AGS is in the middle of MGC‐803 and HGC‐27. Therefore, these three gastric cancer cells, MGC‐803, AGS and HGC‐27, were included in the following analyses.

### 3.4 The effect of sulfasalazine on the proliferation of three cancer cells by CCK‐8 assay

**FIGURE 2:**
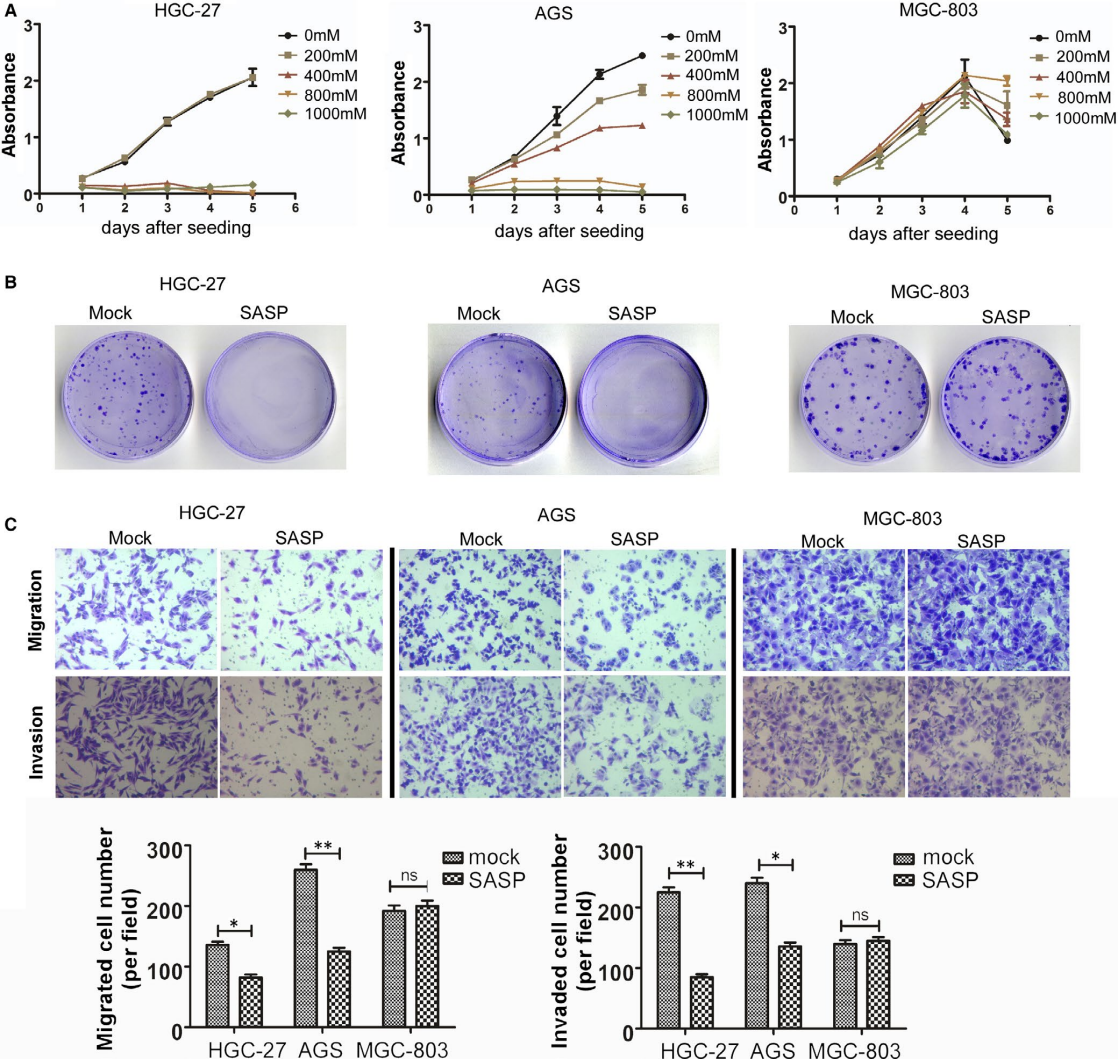
The effect of sulfasalazine on the proliferation, colony formation, metastasis and invasion of gastric cancer. A, CCK‐8 assays revealed the effect of sulfasalazine on HGC‐27, AGS and MGC‐803 cell proliferation. B, Cell colony formation assays were utilized to evaluate the effect of sulfasalazine on HGC‐27, AGS and MGC‐803 cell proliferation. C, Transwell assays were performed to evaluate sulfasalazine's effect on the migration and invasion abilities of HGC‐27, AGS and MGC‐803 cells (*P* < .05)

We utilized CCK‐8 assays to evaluate the impact of the sulfasalazine on the proliferation of three cancer cells. The results of CCK‐8 assays in HGC‐27 cells demonstrated that when the concentration of sulfasalazine higher than 200 mmol L^−1^, the absorbance of the HGC‐27 was dramatically decreased, which indicated that the proliferation of HGC‐27 was attenuated when the concentration elevated (Figure 2A). The results of CCK‐8 assays in AGS demonstrated that the absorbance of AGS was gradually decreased when the concentration of sulfasalazine was elevated, which means the sulfasalazine can attenuate the proliferation of AGS. Nevertheless, there was no significant difference in absorbance in MGC‐803 among different sulfasalazine concentration groups, which implies sulfasalazine may have no impact on the proliferation in MGC‐803.

### 3.5 The effect of sulfasalazine on colony formation ability of three cancer cells by colony formation assay

We utilized colony formation assays to evaluate the impact of the sulfasalazine on the proliferation of three cancer cells. The concentration of sulfasalazine was 800 mmol L^−1^. The results of colony formation assay in HGC‐27 and AGS cells demonstrated that the ability of colony formation in sulfasalazine groups was dramatically decreased when compared with mock groups, which implies sulfasalazine may attenuate the colony formation ability in HGC‐27 and AGS cells (Figure 2B). However, there was no significant difference in colony formation ability in MGC‐803 between the sulfasalazine group and the mock group, which implies sulfasalazine may have no impact on colony formation ability in MGC‐803 (Figure 2B).

### 3.6 The effect of sulfasalazine on migration and invasion abilities of three cancer cells by Transwell assay

We utilized the Transwell assay to evaluate the impact of the sulfasalazine on the migration and invasion abilities of three cancer cells. The concentration of sulfasalazine was 800 mmol L^−1^. The results of the Transwell assay in HGC‐27 and AGS cells demonstrated that the capabilities of migration and invasion in sulfasalazine groups were decreased when compared with mock groups, which implies sulfasalazine may attenuate the migration and invasion abilities in HGC‐27 and AGS cells (Figure 2C). However, there was no significant difference in migration and invasion abilities in MGC‐803 between the sulfasalazine group and the mock group, which implies sulfasalazine may have no impact on migration and invasion abilities in MGC‐803 (Figure 2C).

## 4 DISCUSSION

Gastric cancer remains the fourth most prevalent malignant cancer all over the world and the second leading cause of cancer‐specific death worldwide survival.^16^, ^17^ The incidence of gastric cancer is high in China, with approximately 400 000 newly diagnosed cases every year.^18^ Nowadays, surgical resection remains the mainstay for gastric cancer. Nevertheless, when distant metastasis appears, chemotherapy is required. However, chemotherapy's side effects usually make its administration difficult in some patients, especially those geriatric patients, with comorbidities or decreased general status.^19^ Therefore, alternative therapeutic options are usually and urgently needed.

In this study, we thoroughly compared the expression of xCT and clinical characteristics of gastric cancer. The results demonstrated that higher expression xCT was associated with more advanced gastric cancer, including more T3 and T4 patients and more stage III and stage IV patients. Also, the higher expression of xCT is associated with poor overall survival. Wang et al^20^ performed an IPA analysis of microarray data and founded that the up‐regulation of xCT induced by salubrinal might involve cell death and cell proliferation. They have also found that higher xCT expression in gastric cancer under adjuvant chemotherapy possessed lower progression‐free survival and overall survival than patients with lower xCT expression, respectively. The results mentioned above are consistent with our outcomes.

We have searched and reviewed the previous literature and founded that most studies reported that the expression of xCT is up‐regulated in many kinds of cancer cells. Chen et al^21^ demonstrated that xCT plays an important role in xCT‐dependent cell death in breast cancer cells under glucose deprivation. Also, Ungard et al^22^ demonstrated that the attenuation of xCT in breast cancer cells could delay the onset of bone pain induced by cancer. Horibe et al^23^ showed that up‐regulation of xCT is involved in the process of cisplatin resistance in lung cancer cells. However, the comparison of xCT expression between gastric cancers and normal gastric tissues has not been reported.

In this study, we utilized a conventional drug, sulfasalazine, to evaluate the effects of attenuating the proliferation, colony formation, metastasis and invasion of gastric cancer. Three gastric cancer cells with high, medium and low expression of xCT were selected for the following analyses. CCK‐8 assays revealed that sulfasalazine could attenuate the proliferation of HGC‐27 and AGS. The colony formation assay also revealed that sulfasalazine might attenuate the colony formation ability in HGC‐27 and AGS cells. Plus, the Transwell assays demonstrated that sulfasalazine might attenuate the migration and invasion abilities in HGC‐27 and AGS cells. Shin et al^24^ showed that the up‐regulation of xCT was correlated with cancer stage and progression. Also, they revealed that elevated xCT expression could promote cell proliferation in vitro and tumour growth in vivo, which is consistent with our study. Ji et al^25^ revealed that decreased xCT transport activity by sulfasalazine in xCT overexpressing non‐small cell lung cancer might decrease cell proliferation and invasion both in vitro and in vivo. Fan et al^26^ demonstrated that activation of the Nrf2‐Keap1 signalling accompanied by elevated expression of xCT could promote cell proliferation and resistance to cell death.

In conclusion, higher expression of xCT is associated with advanced tumour stage and poor overall survival of gastric cancer. Sulfasalazine can attenuate the proliferation, colony formation, metastasis and invasion of gastric cancer in vitro. Further study is required to validate our findings.

## CONFLICT OF INTEREST

The authors declared that they have no conflicts of interest in this work.

## AUTHOR CONTRIBUTIONS

**Jinfu Zhuang:** Conceptualization (lead); Data curation (lead); Formal analysis (lead); Funding acquisition (equal); Investigation (equal); Methodology (equal); Project administration (equal); Resources (equal); Software (equal); Supervision (equal); Validation (equal); Visualization (equal); Writing‐original draft (equal); Writing‐review & editing (equal). **Xing Liu:** Conceptualization (equal); Data curation (equal); Formal analysis (equal); Investigation (equal); Methodology (equal); Project administration (equal); Visualization (equal); Writing‐original draft (equal); Writing‐review & editing (equal). **Yuanfeng Yang:** Validation (equal); Visualization (equal); Writing‐original draft (equal); Writing‐review & editing (equal). **Yiyi Zhang:** Data curation (equal); Methodology (equal); Software (equal); Validation (equal); Visualization (equal); Writing‐original draft (equal); Writing‐review & editing (equal). **Guo‐Xian Guan:** Conceptualization (lead); Data curation (equal); Formal analysis (equal); Funding acquisition (equal); Investigation (equal); Methodology (equal); Project administration (equal); Resources (equal); Software (equal); Supervision (lead); Validation (equal); Visualization (equal); Writing‐original draft (equal); Writing‐review & editing (equal).

## Data Availability

Data sharing not applicable to this article as no datasets were generated or analysed during the current study
